# Promoting Children’s Psychomotor Development with Multi-Teaching Didactics

**DOI:** 10.3390/ijerph191710939

**Published:** 2022-09-01

**Authors:** Pietro Luigi Invernizzi, Gabriele Signorini, Marta Rigon, Alin Larion, Gaetano Raiola, Francesca D’Elia, Andrea Bosio, Raffaele Scurati

**Affiliations:** 1 Department of Biomedical Sciences for Health, Università degli Studi di Milano, 20129 Milan, Italy; 2 Sport Faculty, San Antonio Catholic University of Murcia, 30107 Murcia, Spain; 3 Faculty of Physical Education and Sport, Ovidius University of Constanta, 900527 Constanta, Romania; 4 Department of Political and Social Sciences, University of Salerno, 84084 Fisciano, Italy; 5 Department of Humanities, Philosophy and Education, University of Salerno, 84084 Fisciano, Italy; 6 Human Performance Laboratory, Mapei Sport, 21057 Olgiate Olona, Italy

**Keywords:** teaching, styles, motor learning, physical education

## Abstract

This group randomized control trial examined the dose-response effect of varied combinations of linear and nonlinear pedagogy (enriched physical education with specific program led by specialist vs. conventional physical education led by generalist) for improving first-grade children’s motor creativity, executive functions, self-efficacy, and learning enjoyment. We led three physical education classes per group through 12 weeks of combined instruction, based on linear and nonlinear pedagogy: mostly linear (ML; 80% linear, 20% nonlinear; *n* = 62); mostly nonlinear (MNL; 20% linear, 80% nonlinear; *n* = 61); and control (C; conventional teaching from generalists; *n* = 60). MNL improved in (a) motor creativity ability (DMA; 48.7%, 76.5%, and 47.6% for locomotor, stability, and manipulative tasks, respectively); (b) executive functions (working memory and inhibitory control) for RNG task (14.7%) and task errors (70.8%); (c) self-efficacy (5.9%); and (d) enjoyment (8.3%). In ML, DMA improved by 18.0% in locomotor and 60.9% in manipulative tasks. C improved of 10.5% in enjoyment, and RNG task worsened by 22.6%. MNL improvements in DMA tasks, executive functions, and self-efficacy were significantly better than those in C. ML was better than C in DMA task and in executive functions’ task errors. Overall, ML and MNL approaches were more effective than conventional generalist teaching (C), and the MNL combination of 80% nonlinear and 20% linear pedagogy was optimal. We recommend that educators favor the MNL approach.

## 1. Introduction

Physical education (PE) involves practical instructional experiences via body movement, and it represents a significant educational domain for promoting balanced motor, cognitive, and socioemotional learning development in youth. In this way, modern PE favors both specific (motor) and transversal (cognitive and socioemotional) learnings [1,2]. Moreover, PE includes a matrix of learning content from varied disciplinary domains to prepare children for a rapidly changing society that requires them to expand their critical thinking, psychophysical energy, and psycho-emotional capacity to self-manage uncertainty. Although various pedagogical methods for enhancing motor, cognitive, and affective skills through PE have been explored [3], to the best of our knowledge, no studies have examined dose-response effects of varied combinations of linear and nonlinear instructional approaches. Therefore, in this study, we tested the impact of different combinations of linear and nonlinear pedagogy on young children’s motor creativity, executive functions, self-concept, and enjoyment.

Creativity is the general ability to generate new ideas or appropriate outputs in response to problems [4,5], and it comprises both non-cognitive elements (feelings) and cognitive (knowledge) elements [6]. Creativity represents an essential personal asset for children; it expands their ability to live and work in the context of societal change and uncertainty [5,7]. Creativity positively relates to children’s intelligence, executive functions [8], and well-being [9,10].

Motor creativity is a specific aspect of creativity that Wyrick [11] and Tsompanaki [12] have defined as a combination of new perceptions toward motor patterns to solve problems or represent ideas and emotions through movement. Motor creativity has a key influence on children’s motor skill development and has been associated with wide ranging movement outputs, from simple preparatory movement patterns to complex situational expressions [13]. In childhood, motor creativity can be improved by engagement in metaphorical and expressive individual specific motor tasks promoting student’s pursuit fantasy (representing sparks of confidence and competence to engage in novel movements) [14], or by differentiated, constraints-led, reflective, and conscious training based on divergent and convergent thinking [5,14,15]. Cleland [16] found that primary school children benefited more from indirect than direct teaching approaches to improve creativity and critical thinking. Chatoupis [17] suggested that divergent discovery based on flexible and individualized approach is the most appropriate teaching style for developing motor creativity.

Nonlinear pedagogy is an indirect child-centered approach through which the teacher canalizes children’s learning by modifying the task and the environmental and individual constraints rather than by providing demonstrations or instructions [18,19]. From a neurophysiological perspective, these experiential adjustments affect cerebral plasticity favoring creativity [20]. By contrast, linear pedagogy is a direct teacher-centered approach [19,21], wherein learners repeat the task that the teacher or mates demonstrate [19,22]. A linear approach, based on imitation and demonstration, and led by a motivated teacher, can also be a valuable and powerful pedagogic orientation for fostering children’s fundamental motor skills, and cognitive and emotional engaging, at the beginning of the learning process as prerequisite of creative capacities [23]. Combining imitation and emotional participation and practice based on expressive movements, as in the Garçia–Plevin method [24], strongly affects creativity.

The different pedagogical approaches, considering different modalities to balance repetition and changes, stability, and flexibility, can also impact differently on motor and executive functions development [25,26]. For example, both nonlinear and linear approaches positively affect children’s inhibitory control and working memory [20]. Nonlinear pedagogy stimulates inhibitory control and working memory whenever teacher-set constraints require children to inhibit spontaneous conduct or previously used movements actions plans in favor of more effective unusual and novel behaviors to complete the posed assignments [27]. Similarly, until supported by adequate motivation, linear pedagogy stimulates inhibitory control and working memory whenever a new task is repeated many times under the teacher’s or mate’s feedback and corrections or is provided by executive contrasts involving kinesthetic differentiation [28]. By comparing the performed tasks to the expected models, thanks to previous motor knowledge (working memory), children can inhibit incorrect conduct and limit errors [29,30].

Inhibition and working memory development are guaranteed through movement repetitions by *repeating without repeating* [31], as happens in nonlinear pedagogy when children experience new task resolutions or in linear pedagogy when teachers continually vary the task stimulating contextual interference effects [28,32]. When the subjects are too young or inexperienced, or the movement to be learned is too complex, an excessive contextual interference could contrast learning. In these situations, significant and emotional involvement in simple and imitative movement [33] determines the most appropriate stimuli for mental effort and executive functions [34,35]. Zelazo and Carlson [36] highlight the importance of the affective aspects in stimulating hot executive functions which, thanks to a high emotional involvement, can be better reflected in psychomotor integrated development and a creative motor context [37].

Global self-concept, physical self-efficacy, and enjoyment are key factors for promoting motor learning and development because they lead to better compliance with physical activity [38] and motivate children to acquire motor competence and its extension and higher development level constituted by the motor creativity [38,39,40,41,42].

At school, children experience critical periods for developing body self-concept and confidence. Therefore, motor learning at school should provide the tools needed to promote these psychophysical gains. Previous research has shown that a hybrid linear and nonlinear pedagogic approach that engages several teaching styles and promotes vigorous physical activity favors children’s self-efficacy development more than non-integrated practices [43,44]. 

Positive motor experiences carried out through engaging didactics based on mixed and integrated teaching styles represent a rich resource for amplifying the relationship between the different domains of the personality and promoting an optimal psychomotor development [45].

This study aimed to verify the dose-response effect of varied combinations of linear and nonlinear pedagogy for improving first-grade children’s motor creativity, executive functions, self-efficacy, and learning enjoyment. Based on multi-teaching styles and active reflection principles that integrate different teaching strategies to succeed in PE [45], we investigated three teaching pedagogies: (a) 80% linear and 20% nonlinear, (b) 20% linear and 80% nonlinear, and (c) a conventional generalist teaching style. The linear and nonlinear combinations were intended to address varied student fantasy and teacher manipulating constraints to enhance psycho-motor development. However, despite contrasting unbalanced pedagogy proportions, we hypothesized that both approaches have substantial beneficial effects on mentioned variables and that both enriched pedagogies of this study are significantly superior to the control approach of generalists’ conventional pedagogy.

## 2. Materials and Methods

### 2.1. Participants

The study protocol was approved by the University of Constanta Ethics Committee and conformed with the Declaration of Helsinki (1964). Before beginning the study, we explained the procedures and goals to the schools’ principals, to the children, and to their parents and guardians, and, prior to any child’s involvement in the study, we obtained advanced informed written consent from all parents/legal guardians and advanced assent from all children.

**Figure 1:**
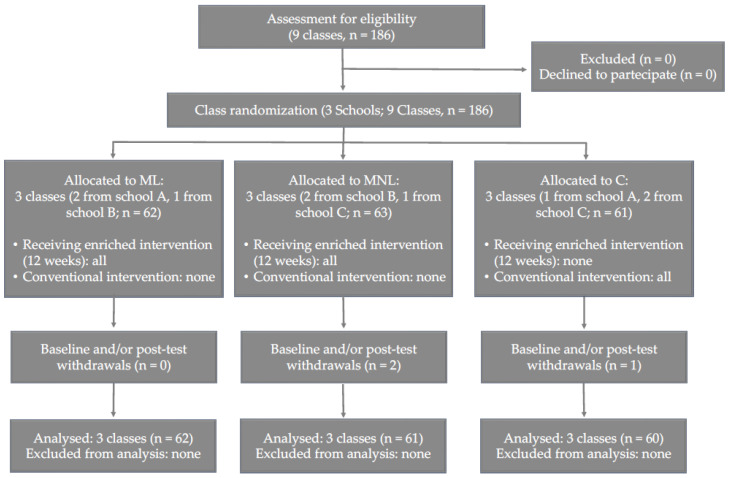
Study flow diagram of the class-randomized trial.

We recruited 186 first-grade children from three primary schools. To be included, children had to be attending PE in school classes twice a week (in total, two hours per week), as determined from checking the school registers, and no child could have any neurologic disorders or disabilities. Three children withdrew at baseline and/or post-testing, then the analysis was performed on the remaining 183 participants (M age = 6.5, *SD* = 0.7 years; M height = 1.18, *SD* = 0.07 m; M weight = 21.4, *SD* = 2.5 kg; M body mass index [BMI] = 15.3, *SD* = 1.3 kg/m^2^). Following the CONSORT guidelines [46], Figure 1 reports the study flow of the class randomized trial, from enrolment to allocation and final sampling.

### 2.2. Experimental Design and Procedure

In this class-randomized control trial, children were attending first-grade in one of nine different primary school classes with no differences in settings, location, or socioeconomic origin (middle social environment, as deduced from the three-year plan of the educational offer of schools involved it the study). The data controller conducted a random drawing from closed envelopes to determine to which of three instructional methods in each class was allocated. During the 12 weeks of intervention, each group’s PE classes were led using one of three approaches: (a) a mostly linear pedagogy: multi-linear approach with 80% linear and 20% nonlinear teaching (ML: *n* = 62; 43.5% boys, 56.5% girls; *M* age = 6.6, *SD* = 0.8 years; *M* height = 1.19, *SD* = 0.08 m; *M* weight = 21.3, *SD* = 2.4 kg; *M* BMI = 15.1, *SD* = 1.7 kg/m^2^); (b) a mostly nonlinear multi-linear approach with 20% linear and 80% non-linear teaching (MNL: *n* = 61; 50.8% boys, 49.2% girls; *M* age = 6.4, *SD* = 0.7 years; *M* height = 1.18, *SD* = 0.08 m; *M* weight = 21.3, *SD* = 2.8 kg; *M* BMI = 15.2, *SD* = 1.0 kg/m^2^); and (c) a control condition involving a standard teaching approach as taught by generalist teachers (C: *n* = 60; 51.6% boys, 48.4% girls; *M* age 6.3, *SD* = 0.5 years; *M* height = 1.18, *SD* = 0.06 m; *M* weight = 21.6, *SD =* 2.3 kg; *M* BMI = 15.6, *SD* = 1.2 kg/m^2^).

**Figure 2:**
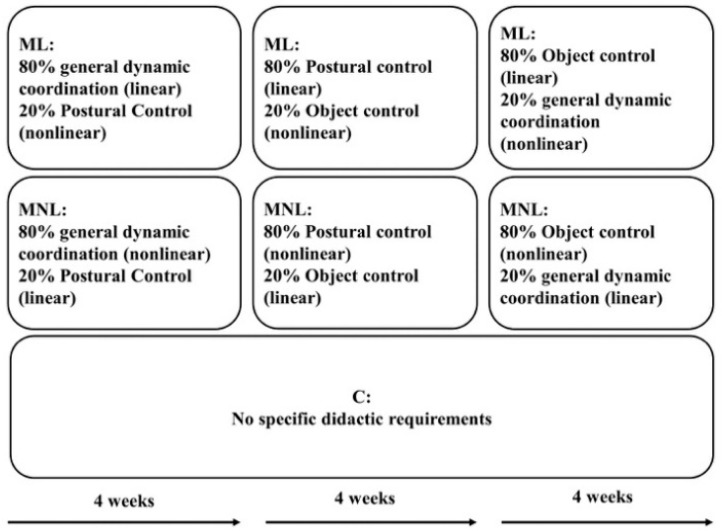
Periodization of PE contents. ML = multi-teaching approach emphasizing linear teaching; MNL = multi-teaching approach emphasizing nonlinear pedagogy; C = control (conventional teaching by generalists).

In the country where this study was performed (Italy), primary school children are taught by two elementary schoolteachers (generalists), one for humanities and one for scientific disciplines, including PE. Outsourcing projects sometimes allows PE specialists to lead PE classes or support the generalist teachers. This study took advantage of running outsourcing projects to recruit specialists for experimental groups and generalist teachers’ availability to participate in the research as a control group. Therefore, two PE specialists from the outsourcing projects taught the ML and MNL classes, while a generalist teacher led the C classes (two primary school teachers participated, one led two classes). Before beginning the study, the two PE specialists attended three two-hour training sessions with the research team on specific guidelines to follow during the PE lessons. Figure 2 synthesizes the organization and content of the PE lessons (for ML, MNL, and C). Details about pedagogical principles guiding enriched PE programs Ní Chróinín, et al. [47] are provided in the Appendix A.

The generalist teachers in the control group (C) received no specific instruction. Participating children did not know to which enriched physical education experimental group they belonged (ML or MNL).

Children’s motor creativity (movement divergency), executive functioning, self-efficacy, and enjoyment were measured through specific testing procedures (detailed below) before and after the 12 weeks of intervention during curricular PE hours (see Figure 2). Children’s motor creativity measures originated from analyzing testing session video recordings; executive functioning was measured during testing; surveys assessed self-efficacy and enjoyment. It must be noted that, during the study, the generalist schoolteachers expressed interest in detecting any acute effect on the executive functions attributable to teaching approaches on possibly altering student’s participation in the classes scheduled immediately after the PE session [48]. Hence, even if possibly entailing limitations, the *participatory action research* principles [49] were applied to respond to context-specific needs and produced knowledge and direct practical actions for promoting effective strategies in the school context. Therefore, the post-intervention executive functions survey procedure was adjusted, and the testing session was conducted within one hour of the end of the last PE practice.

Physical activity was measured to check that participants did not alter the amount of physical activity during the experimental period to avoid unexpected changes that might introduce a bias factor in the analysis on post-intervention results. To check MNL, ML, and C teaching styles, PE lesson samples were video recorded and analyzed.

### 2.3. Testing 

#### 2.3.1. Divergent Movement Ability Tasks (DMA)

Divergent movement ability (DMA) represented aspects of movement creativity such as fluency and flexibility, and these tasks were explicitly designed to measure movement divergence in PE [16]. The DMA tasks were designed to assess locomotor, manipulative, and stability abilities. In the locomotor task, children performed as many movement patterns as possible within an area presenting different objects (suspended rope, hoola-hoop placed on three cones, gymnastic mattress, and cones). In manipulative tasks, children were invited to play with a ball as many ways as possible. They played in a delimited area that included an available wall. In the stability task, children made as many balanced shapes as possible on a bench surrounded by four gymnastic mats. Each task (locomotor, manipulative, and stability) was performed for one minute and 30 s and repeated twice, for a total of nine minutes. A 2-min rest was provided after each task. Children received verbal encouragement from the experimenter every 30 s, but no suggestion or facilitation was given. For each DMA task, the different solutions participants performed (fluency and flexibility behaviors) were separately recorded onto an observation sheet by two skilled observers who analyzed the testing session video recordings. Children were tested individually. A third investigator coded and assigned the final score only for those behaviors on which the two observers agreed.

#### 2.3.2. Random Number Generation (RNG)

Random Number Generation (RNG) is a cognitive test that evaluates executive functions [50]. On this task, participants were asked to generate a random sequence of numbers, saying a number from 1 to 10 every 1.5 s, 70 times, following a metronome set at 40-bpm [51]. The numbers were recorded in a notebook and the final score was calculated by applying the Towse and Cheshire [50] formula. Lower RNG scores represented better performance. Regarding RNG errors, mistakes (i.e., numbers other than numbers from 1 to 10) were considered invalid (not synced with the metronome beats), and missing answers were considered violations. For data analysis, we considered two indices: the random number generation (RNG task) that was used to evaluate working memory and the random number generation violations (RNG errors) that were related to both working memory and inhibition capacity [50,52].

#### 2.3.3. Physical Activity Questionnaire for Children (PAQ-C)

The Physical Activity Questionnaire for Children (PAQ-C) by Crocker, et al. [53] assesses moderate to vigorous physical activity performed by children in the seven days preceding its administration [54]. It is a self-report instrument that is administered by survey, and it is comprised of 10 items; nine items calculate the activity scores using a five-point Likert-type scale; and a tenth item asks whether sickness or other events interrupted regular physical activity during the week. Children were skilled enough in reading to understand the questionnaires. However, parents were asked to give them support in responding.

#### 2.3.4. Physical Self-Efficacy Scale (PSES)

The Physical Self-Efficacy Scale (PSES) by McAuley and Gill [55] is a valid and reliable tool to assess general self-perception of physical motor abilities’ self-efficacy, such as strength, speed, and coordination [56]. The researcher ascertained that all participants fully understood each question and freely responded, reassuring them that no responses were considered correct or wrong. Scores ranged from 1–24, with higher scores indicating a higher perception of self-efficacy. A skilled experimenter who did not know the participants’ group allocation helped children complete the questionnaire, if needed.

#### 2.3.5. Physical Activity Enjoyment Scale (PACES)

We assessed the children’s enjoyment with the Physical Activity Enjoyment Scale (PACES) [57], which is comprised of 16 items that measure children’s positive involvement in an activity using a 5-point Likert-type scale (1 = “Disagree a lot” to 5 = “Agree a lot”). Nine items are positively expressed (“I enjoy it”; “It gives me energy”), and seven negatively (“I feel bored”; “It frustrated me”). In positively expressed items, higher scores indicate higher enjoyment; in negatively expressed items, high enjoyment corresponds to lower scores. The final score was the mean value of all items. An experimenter who did not know the participants’ group allocation supported children during survey completion.

### 2.4. Teaching Approaches

ML and MNL enriched PE classes were designed under different combinations of linear and nonlinear pedagogy, respectively promoting student fantasy pursuits, and teacher manipulating constraints, while the control group (C) maintained the usual practice. ML specialist teachers based 80% of their teaching on linear pedagogy and the remaining 20% on nonlinear; MNL specialist teachers did the opposite (80% nonlinear and 20% linear). Since no previous studies defined dosages of multi-teaching styles, these percentages were chosen as a starting point to assess the correct styles’ percentage integration that could guarantee suitable effects with first-grade children.

Generalists taught C classes without any specific requirements; generalist schoolteachers mostly use a direct, prescriptive style (command) and learner-initiated activity (free play) for teaching PE [45]. To verify this and ensure that the conventional PE of the control group activity differed from the ML and MNL, and to check the teacher’s pedagogic approach, we analyzed video recordings of three lessons per group throughout the intervention (as later described) for further analysis at the end of the experimental period.

The two weekly hours of curricular ML and MNL physical activity across the 12-week intervention were principally designed to promote motor creativity [58], the highest level of the psychomotor domain, assuming that motor proficiency and effective learning of fundamental motor and play skills were prerequisite [26,59,60,61,62]. Additionally, enriched physical activity further promoted executive functions, self-efficacy, and enjoyment. ML and MNL activities were structured under a didactic progression of four blocks of six lessons each: two structured (entirely planned by the research team), one quasi-structured (partially planned by the research team, partially by the specialist teachers), and one free block (entirely planned by the specialist teachers). At the end of each block, the research team held meetings with the specialist teachers to collect feedback, summarize the advances, check the intervention’s appropriateness, and revise and adapt the following lessons, if necessary. To prevent or limit as much as possible children’s dispersive reflections and time wasting, MNL and ML teachers were required to prioritize the time devoted to practice, restricting the number of interruptions for reflection, and answering a maximum of two questions per interruption.

#### 2.4.1. Multi-Teaching Enriched Approach Emphasizing Linear Teaching (ML)

In linear pedagogy teachers typically model what students should imitate. This method is characterized by allegorical-oriented imagery promoting fantasy during activities with children. In the present study, the teacher or a teammate demonstrated the exercise to give adequate information (modeling) and allowed the performers to reproduce the motor pattern correctly. In the predominantly linear approach, the proportion of linear pedagogy devoted to stimulating the children’s fantasy accounted for 80%, while the remaining 20% of teaching time was based on nonlinear pedagogy, as described below.

A teacher demonstration was given first, then children reproduced the motor pattern. Verbal instructions described how to correctly perform the requested motor pattern, and questions were posed to help motor learning. Feedback was given only to correct relevant mistakes or when the motor pattern was no longer recognizable as performed. The practice was repeated several times to consolidate learning. Attention was focused on *how to do* the motor task. An example of structured practice based on linear pedagogy is reported in the Appendix B. An example of the structured practice prevalently based on linear pedagogy, as used in teaching ML.

#### 2.4.2. Multi-Teaching Enriched Approach Emphasizing Nonlinear Teaching (MNL)

Nonlinear pedagogy explores creativity and originality. Therefore, specialist teachers avoided modeling and demonstration in MNL, and their verbal instructions were minimal and not prescriptive. Specialist teachers had to build adequate environments to manage the tasks and promote active new actions from the students. In the predominant nonlinear approach, nonlinear pedagogy devoted to manipulating constraints comprised 80% of the teaching, with the remaining 20% of teacher time involved in promoting fantasy through modeling and prescriptive teaching (linear approach).

The teachers’ support to students during predominant non-linear teaching was based on questioning to better stimulate children to discover different and varied solutions to the required task; consequently, children’s behaviors were thoroughly observed to address the supporting process. The motor experience depended on the situational constraints set by the teachers to create movement challenges through tasks and environment variations. Children’s attention was directed to *what* to do (external focus attention) instead of *how* to do the tasks. Examples of MNL are given in the Appendix C. An example of the structured practice prevalently based on nonlinear pedagogy, as used in teaching MNL.

#### 2.4.3. Control Condition of Conventional Pedagogy (C)

As previously reported, the C group physical activity was led by the primary school generalist teachers of the three classes composing the control group. These teachers were not requested to use any specific teaching approach or style. Instead, they were asked not to alter their usual interventions, which were assumed to correspond to a prescriptive style (command) and learner-initiated activity (free play), as reported in the literature [45]. Evidence of a conventional PE approach was examined by analyzing the lesson videos and the differences from specialist approaches.

### 2.5. Video Analysis of the PE Lessons

One lesson per teaching block (structured, quasi-structured, and free; see Section 2.4) was randomly selected for video recording to be used for subsequent analysis to assess the salient features of ML or MNL lessons. In addition, lessons for the C group were also video recorded at three different time points (at the beginning, middle and the end of the intervention period).

Two PE experts not directly involved in the study and who were unaware of the participants’ group assignment analyzed and evaluated the recordings, using the Instrument for Identifying Teaching Styles (IFITS) by Curtner-Smith [63]. They observed the teaching styles in the lessons and assessed them to verify the correct intended application of the experimental design. Production styles as learner-initiated activity and guided discovery teaching styles were considered nonlinear pedagogy, while the reproduction teaching style was considered linear [64]. The PE experts were experienced in data collection and in operating code instruments, and they had previously completed a four-hour training to revise the procedure thoroughly. Resting, inaction, and reflection times were further analyzed to assess the effects of each teaching approach on children’s activity. These parameters depict the children’s activity features as they related to the specialists’ structured MNL and ML teaching approach and the standard generalist approach.

### 2.6. Statistical Analysis

Statistical analyses were performed using XLSTAT 12.3.01 (Addinsoft, New York, NY, USA) and the Statistical Package of the Social Sciences (SPSS, version 20.0, IBM Corp. Armonk, NY, USA) software. We used the Shapiro–Wilk test to assess the data’s normal distribution. Non-parametric analyses were then performed because the data were not normally distributed. We checked the reliability of the DMA and RNG measurements’ using a non-parametric version of the intra-class correlation coefficient (ICC). We used the Wilcoxon test to analyze the within-group effects induced by teaching approach and compare the data collected before and after the intervention. We applied the Kruskal–Wallis test to assess group homogeneity before the intervention. The post-intervention data of variables that confirmed homogeneity at baseline were compared by the Kruskal–Wallis test and Dunn’s post-hoc test with Bonferroni correction to detect changes ascribed to different treatments. The treatment effects on data that violated the homogeneity assumption and differed at baseline were investigated by comparing the deltas (after-before) with the Kruskal–Wallis test and Dunn’s post-hoc test with Bonferroni correction.

We calculated the effect size by epsilon squared, according to Tomczak and Tomczak [65]. The corresponding ES thresholds for *very small*, *small*, *moderate*, and *large* effects were classified as <0.02, 0.02 to 0.13, 0.13 to 0.26, and >0.26, respectively [66]. Moreover, an ICC assessed the intra- and inter-rater reliability in PE lesson video analysis. Statistical significance level was set at *p* < 0.05 for all comparisons.

## 3. Results

All the three groups were found homogenous for age (*p* = 0.304), weight (*p* = 0.925), height (*p* = 0.612), and BMI (*p* = 0.573). DMA and RNG measurements performed before the intervention were reliable (locomotor task: ICC = 0.974, *p* < 0.0001; stability task: ICC = 0.995, *p* < 0.0001; manipulative task: ICC = 0.966, *p* < 0.0001; RNG task: ICC = 0.937, *p* < 0.0001; RNG errors: ICC = 0.957, *p* < 0.0001).

Changes that occurred within each group after 12 weeks of intervention are reported in Table 1. MNL significantly improved in all DMA tasks by 48.7%, 76.5%, and 47.6% in locomotor (*p* < 0.0001), stability (*p* < 0.0001), and manipulative tasks (*p* < 0.0001), respectively. Moreover, although ML significantly improved locomotor and manipulative tasks by 18.0% and 60.9%, respectively (*p* = 0.001 and *p* < 0.0001), the 8.9% change in the stability task was not statistically significant (*p* = 0.068). In contrast, the C group did not significantly improve, and post-intervention performance was comparable to baseline (locomotor task: *p* = 0.520; manipulative task: *p* = 0.734; stability task: *p* = 0.895).

MNL significantly improved RNG task scores, which decreased by 14.7% (*p* = 0.028); RNG errors were reduced by 70.8% (*p* < 0.0001). In contrast, ML showed no changes from baseline (RNG task: *p* = 0.400; RNG errors: *p* = 0.067), while C significantly worsened by 22.6% in the RNG task (*p* = 0.001), with no changes from baseline for C group RNG errors (*p* = 0.054).

Comparing questionnaire scores before and after intervention showed significant differences in PSES and PACES for the MNL group, which improved by 5.9% (*p* = 0.008) and 8.3% (*p* = 0.004), respectively; the C group PACES improved by 10.5% (*p* = 0.025).

**Figure 3:**
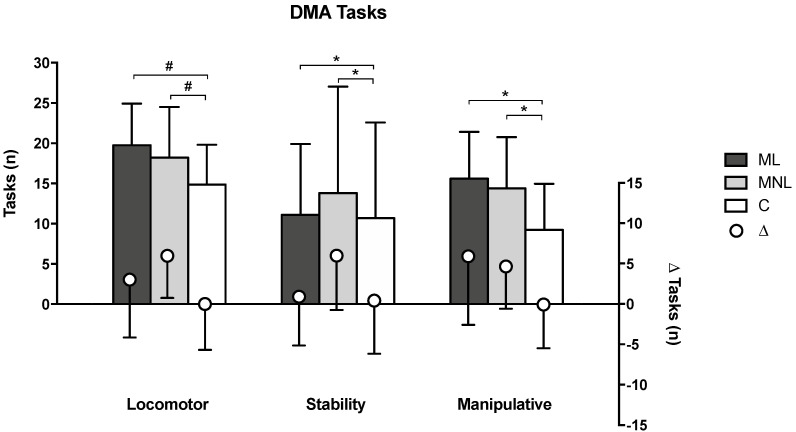
Effects of different teaching approaches on divergent movement ability (DMA) after 12 weeks of intervention. ML = multi-teaching approach emphasizing linear teaching; MNL = multi-teaching approach emphasizing nonlinear pedagogy; C = control (conventional PE teaching by generalists); Δ = within-group post-intervention changes compared to baseline. Significant values are shown: # = *p* < 0.05 (analysis performed on ∆ after-before intervention because homogeneity at baseline was not met); * = *p* < 0.05 (analysis performed on post-intervention measures because baselines were homogeneous).

**Figure 4:**
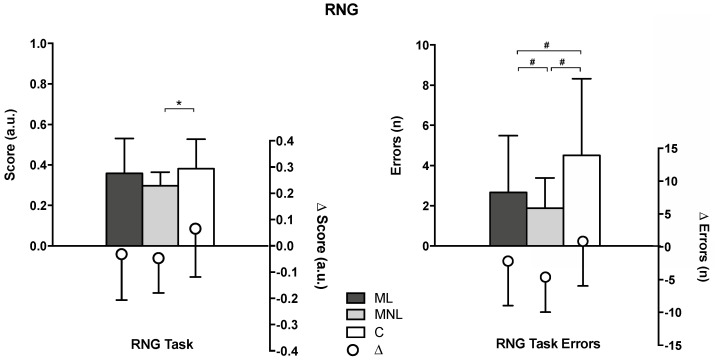
Effects of different teaching approaches on random number generation (RNG) after 12 weeks of intervention. ML = multi-teaching approach emphasizing linear teaching; MNL = multi-teaching approach emphasizing nonlinear pedagogy; C = control (conventional PE teaching by generalists); Δ = within-group post-intervention changes compared to baseline. “a.u.” = arbitrary units. Significant values are shown: # = *p* < 0.05 (analysis performed on ∆ after-before intervention because homogeneity at baseline was not met); * = *p* < 0.05 (analysis performed on post-intervention measures as baselines were homogeneous).

The between-group comparisons showed that ML and MNL had comparable results in DMA tasks, while C had significantly lower DMA scores than ML or MNL (Table 2, Figure 3). However, after intervention, RNG task scores significantly differed only between C and MNL, while RNG task errors were higher in C compared to ML and MNL, with MNL showing the best outcomes (Figure 4).

**Figure 5:**
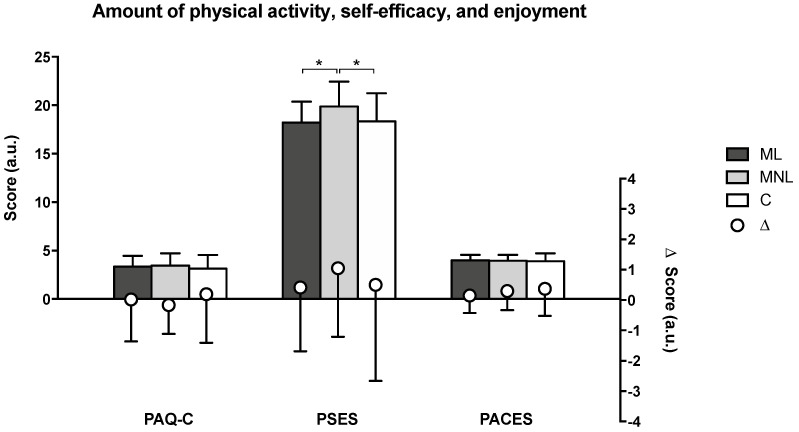
Effects of different teaching approaches on the amount of physical activity, self-efficacy, and enjoyment measured by questionnaires (PAQ-C, PSES, PACES) after 12 weeks of intervention. ML = multi-teaching approach emphasizing linear teaching; MNL = multi-teaching approach emphasizing nonlinear pedagogy; C = control (conventional PE teaching by generalists); Δ = within-group post-intervention changes from baseline. “a.u.” = arbitrary units. Significant values are shown: * = *p* < 0.05 (analysis performed on post-intervention measures because baselines were homogeneous).

Figure 5 and Table 2 show the PAQ-C, PSES, and PACES questionnaire results. PAQ-C and PACES showed no differences by teaching approach, whereas MNL obtained the best PSES scores, significantly higher than those of ML or C.

### PE Lessons’ Video Analysis

The ICC showed intra-rater reliability values of 0.90, 0.95, 0.91, and 1.00 for resting time, reflection time, inaction time, and total time activity, respectively. Inter-rater reliability was 0.80, 0.92, 0.93, and 1.00 for resting time, action time, reflection time, and total time, respectively.

The intra-rater reliability analysis for adopted teaching styles were 0.82 for linear teaching styles and 0.85 for nonlinear teaching styles in ML; 0.93 for nonlinear teaching styles and 0.90 for linear teaching styles in MNL; 0.99 for prescriptive approach without imitation and 1.00 for free play in C. Inter-rater reliability was: 0.71 for linear teaching styles and 0.81 for nonlinear teaching styles in ML; 0.89 for nonlinear teaching styles and 0.90 for linear teaching styles in MNL; 0.97 for prescriptive approach without imitation and 1.00 for free play in C.

The specialist teachers respected the assigned teaching method (Table 3, Figure 2), while generalist teachers primarily engaged children with free play, and to a limited extent, a command teaching style. Even if free play were considered a nonlinear approach and command teaching was considered linear, the video recordings documented their essential traits. Free play used by generalist teachers resembled entertainment more than a direct-to-learning approach. Moreover, unlike ML, C’s command teaching was limited to mere reproduction of movements and lacked imitation exercises based on allegorical-oriented imagery and reflective action. Moreover, generalist teachers allowed children more resting time than specialists.

## 4. Discussion

This study investigated different teaching approaches’ dose-response effects on motor creativity, executive functions, self-efficacy, and enjoyment associated with teaching physical activity to first-grade children. The enriched PE based on multi-teaching approaches integrating linear and nonlinear pedagogy, and the pedagogical principles as reported in Appendix A were better than the conventional PE approach used by generalist schoolteachers. Linear pedagogy was based on the cognitive theory (central theory) and on deliberate practice, in which the learning referred to a specific model proposed by a metaphoric image that learners tried to understand (through demonstration) and imitate [20]. Nonlinear pedagogy was based on the dynamic ecological approach (peripherical theory), in which the learners were stimulated to explore the learning environment and find one or more personal solutions [20,67]. After 12 weeks of practice, the MNL approach, with 20% linear and 80% nonlinear pedagogy induced improvement in nearly all measured variables in both within and between comparisons. Although MNL seems preferable, the ML teaching approach, with 80% linear and 20% nonlinear styles surpassed the C condition as well.

The improved motor creativity and executive functions in the two multi-teaching enriched groups, measured by DMA and RNG tasks, represent this study’s most notable outcomes (Table 1, Figure 3 and Figure 4). RNG tasks have been shown to reflect children’s executive functions, a measure of motor creativity task and a feature of divergent and critical thinking [26]. Executive functions are demonstrated to be essential for mental and physical health [68]. They are involved in multi-teaching styles, where the interaction of productive and reproductive styles stimulates inhibition and memory updating, which are required to excel in RNG tasks [25]. The results of motor creativity and executive functions presented mostly medium effect sizes, likely to the literature that in similar studies reported medium to large effect sizes [26,69,70]. Moreover, the results align with those by Pesce, Masci, Marchetti, Vazou, Saakslahti, and Tomporowski [51], highlighting that integrating motor and cognitive tasks is essential for improving children’s creative motor skills. 

Moy, et al. [71] asserted that nonlinear-based approaches increase intrinsic motivation, self-determination levels, and enjoyment. This study’s within-group outcomes (before-after) confirm the approaches’ positive effects on enjoyment [27]. Of note, the MNL teachers’ autonomy established all affordances inviting children to play with the body or unexperienced devices, which had a significant impact on enjoyment and self-efficacy because they led to emotional empowering [72]. Furthermore, even if the effect sizes from the present analysis differed little from the considered literature (medium vs. large effect size), this confirms what was expressed by other authors [72]. By contrast, direct styles such as ML, even if based on fantasy (enhancing imagination) and on exercise imitation (enhancing fundamental motor skills), which are prerequisite of motor creativity, are not sufficient and could limit children’s free movements and, therefore, free expression [73]; hence, the need to dose a correct percentage of approaches to create a proper stimulation of the enjoyment. Pedagogic approaches need to be supported by a mastery climate stimulation, which implies allowing children to freely select how to interpret a task’s resolution based on personal abilities, as in the nonlinear pedagogy. Such a hybrid approach emphasizing MNL pedagogy improves self-efficacy, fundamental motor skills (expressed in the DMA as extension of the meaning of motor competence [42]), and motivational climate, which are necessary prerequisites for maintaining positive lifestyles through the lifespan based on sound physical practices [41,74].

In the beginning, a linear pedagogy by imitative approach may favor early children as it permits acquiring a wide range of fundamental motor skills without complex and extreme creative efforts [60,62,75]. The lack of changes in ML self-efficacy and enjoyment in the within-group (before-after) and between-group (ML-MNL-C) comparison may be explained by the children’s different engagement when practicing under a linear rather than a nonlinear approach. Nonlinear pedagogy specifically considers functional effort reliant on the motor task associated with involvement, ability, and physical and mental conditions during task execution [76]. By contrast, linear pedagogy considers a nominal effort reliant on the motor task, which exclusively refers to the task characteristics and is not necessarily aligned with individual capacities [76], and, therefore, does not offer the same chance of success to all children. 

Finally, the findings showed that, similar to MNL, C experienced high enjoyment (as it can be observed in the within-group comparison). As previously reported in the literature [45], free play used by generalist teachers may raise children’s enjoyment, but is not sufficient to promote motor learning, possibly because the activity resembles entertainment and fun, rather than learning-oriented games. As expected, specialist teachers provided the planned dose of linear and nonlinear teaching styles (Table 3). By contrast, having no specific experience in PE, generalist teachers primarily provided free play and command teaching styles. Excessive involvement in free and non-controlled play is not sufficient to create competencies and learning, which explains the lower DMA and RNG results in C compared to ML and MLN (Table 1, Figure 3 and Figure 4) [77].

Moreover, generalist teachers allowed children more resting time (28.3%) than specialists (ML 4.3%, MNL 8.5%) and did not spend time reflecting with them. As reported in previous research [45], generalist teachers lack specific PE competence. Therefore, they are more likely to let children freely play or to teach by command than to apply more elaborate teaching styles devoted to improving the students’ critical thinking, because managing the latter is more complicated [78].

Additionally, the C findings denote worsening cognitive function indicators measured by RNG (Table 1), which is worthy of attention and further investigation. Montessori and Lamparelli [79] pointed out that games should not be considered unnecessary and useless pastimes without achievements or even fostering inattentiveness and devolution. Games should be conceived as *exercise games* and deliberate play, which are significant, structured, and finalized to develop children’s creative mind [15,79]. Rather than to the games themselves, Montessori’s critical comments were instead directed to teachers’ occasional misinterpretation and inappropriate use of playing. Excessive involvement in free and non-controlled play lacking reflection on behaviors might induce hyperactivity, compulsive actions, self-control failures, and attention deficits [80,81]. As in non-controlled play, excessive reactivity to stimuli and states of intense emotional arousal can lead to children’s dysregulation [82]. A lack of clear, structured, and direct targets and rules can affect participants’ executive functions. Schoolteachers should consider this in planning educational programs, possibly avoiding topics requiring great attention immediately after PE classes involving children with free and non-controlled play.

The best results of MNL compared to ML in the within comparison (Table 1) can be explained by the rationale that guided selecting the activities to include in the practice (see Appendix B and Appendix C). Until 6 to 7 years of age, similar to the present study’s participants, children are still developing neurocognitive processing based on combining cognitive and fundamental motor skills. They have not yet adequately consolidated the temporal and spatial features; consequently, they have a primarily static body’s mental representation, with subsequent difficulties in perceiving themselves in dynamic situations [83]. Thus, MNL’s practice integrates mental representation and body perception better than ML’s static exercises, which are based on more minor stimulating cognitive functions, such as imitation and reproduction, and not on problem solving. More than ML, MNL’s broader and more diversified approach may elicit contextual interference processes on the proposed practice [84,85,86,87], which fostered and improved static body perception [88]. By contrast, in this transitional age, dynamic tasks can only be acquired in holistic and more unconscious ways, implying that the nonlinear approach’s higher and more appropriate contextual interference does not result in learning efficacy that is superior to that of linear pedagogy [14,89]. 

According to the World Health Organization, moderate to vigorous physical activity has potential effects on physical, cognitive, and psycho-emotional domains [90]. However, the PAQ-C analysis confirmed that our participants engage in a comparable amount of physical activity during the experimental period, which was unchanged and did not differ between groups. This corroborates that the response to PE classes on the variables measured originated from the teaching pedagogies and dosage dissimilarities rather than different volumes of physical activity. In addition, it is reductive to consider only quantitative aspects of physical activity in children and adolescents [60].

Given this, our outcomes partially agree with previous studies’ findings that nonlinear approaches are a powerful pedagogy for children, such as Cleland [16], who found that nonlinear teaching is adequate to acquire motor skills and significantly surpasses traditional teaching methods and linear pedagogy. Similar to MNL, using fantasy, metaphoric imagery, and reflective action in the imitation exercises possibly promoted ML’s motor creativity and executive functions more than the less structured approach used with C (Figure 3 and Figure 4). At our participants’ age (6 to 7 years), corresponding to the transition between early childhood (2 to 6 years) *preoperational/symbolic thinking age* and late childhood (7 to 9 years) *early concrete operational* stages [14,91], children operate in an actual state with modified meaning, whose imaginative component is essential to comprehending reality [92]. Noticeably, even if the C group’s activity resembled that of ML for the prescriptive teaching style, they substantially differed. ML was thoroughly designed and managed to stimulate children’s imitation, imagination, and fantasy (see Appendix B), while C’s linear approach was limited to letting the children move.

### 4.1. Ethics of Participatory Action Research with Children

In line with ethics based on how the children approach the research in a participatory action research method [93] and in respect of The UN Convention on the Rights of the Child (UNCRC) [94], the present research was designed to develop a fun and child-friendly methodology, and to ensure children’s interest and natural approach to the research as part of the formative scholastic process. Specifically, the goal of the education (Art. 29: *education must develop every child’s personality, talents and abilities to the full*) and the fairest interest of the child (Art. 3: general principle) have been considered. In this way, in involving children in the research, we contemplated:

- The research context (classrooms and school gyms): that they were a safe and usual known space of the school;
- The informed consent addressed to children: that it was presented with simple and straightforward language and with metaphorical images and drawings;
- The researchers charged to give explanations to the children: that they were long-experience PE teachers, skilled in establishing a good relationship with the children involved in the study, reassuring them about the pleasantness of the PE lessons contents and the easiness of tests and questionnaires not having right or wrong executions and answers, allowing everyone “to doing what he can, not what he cannot” [95].

### 4.2. Limitations

The video analysis of the lesson samples showed that generalist teachers’ activities differed from the specialists in duration and content, with the C lessons about 25% shorter than MNL and ML lessons. It is possible that the reduced activity time may have affected the C results; therefore, these results must be interpreted with caution. However, the poor quality of the generalist interventions compared to the more qualitative structured enriched approach was confirmed.

A further limitation of the study is that the randomization was conducted on classes, not participants. This was due to organizational constraints (the impossibility of altering the study’s class composition in the schools involved in the study). In addition, each teacher’s personality characteristics may have affected the results. Indeed, we could not dispose of teachers who could lead following all approaches considered in this study (ML, MNL and C) due to the same issue concerning the class composition. 

The last limitation of this study is the lack of enrollment in a register for randomized control trials.

## 5. Conclusions

The early primary school children who participated in this study benefited from the two types of enriched PE multi-teaching style approaches based on different styles and active reflection led by specialists in exercise science. These approaches produced a more appreciated and successful physical education experience and are more productive than the non-specific and conventional PE approach used by generalist schoolteachers.

Our findings suggest that in first-grade children of middle socio-cultural status, a mix of 80% nonlinear and 20% linear is the best didactic approach in improving children’s cognitive, psycho-emotional, and creative motor function.

The outcomes of the present study lead to new directions of research aiming to consolidate psychomotor development and suggest shifting from choosing between linear or nonlinear teaching styles to exploring new insights into the integrated motor learning process. The learning process’s complexity should consider a multi-teaching style approach that can be dosed and adapted to learners’ specifics and different socio-cultural contexts [45]. Therefore, the critical point is finding the correct dose of contextual interference to implement, because worsened learning can occur when presenting stimuli that are too complex [96,97]. Too complex and varied tasks may confuse beginners [84,87], but some must be preserved since dose-response aspects, such as cognitive stimuli levels and response quality, are determinant features in enriched PE and holistic development [35]. Future studies should explore different dose-response percentages of style integration.

## Figures and Tables

**Table 1:** Within-group, before–after comparisons of teaching approach effects on critical thinking, executive functioning, physical activity, self-efficacy, and enjoyment.

Variables	ML			MNL			C		
	Before	After	Δ%	Before	After	Δ%	Before	After	Δ%
DMA Locomotor task (*n*)	16.7 ± 8.0	19.8 ± 5.2 *	18.0	12.3 ± 3.7	18.2 ± 6.3 *	48.7	14.9 ± 5.1	14.9 ± 4.9	−0.1
DMA Stability task (*n*)	10.2 ± 10.3	11.1 ± 8.8	8.9	7.8 ± 7.9	13.8 ± 13.2 *	76.5	10.3 ± 10.1	10.7 ± 11.9	4.1
DMA Manipulative task (*n*)	9.7 ± 6.5	15.6 ± 5.8 *	60.9	9.8 ± 5.2	14.4 ± 6.4 *	47.6	9.3 ± 5.4	9.2 ± 5.7	−0.7
RNG task (a.u.)	0.39 ± 0.2	0.36 ± 0.1	−7.7	0.34 ± 0.1	0.30 ± 0.1 *	−14.7	0.32 ± 0.1	0.38 ± 0.1 *	22.6
RNG task errors (*n*)	4.8 ± 6.0	2.7 ± 2.8	−43.7	6.5 ± 5.5	1.9 ± 1.5 *	−70.8	3.7 ± 6.8	4.5 ± 3.8	21.6
PAQ-C (a.u.)	3.3 ± 1.4	3.4 ± 1.1	0.6	3.6 ± 1.2	3.4 ± 0.3	−4.3	3.0 ± 1.4	3.2 ± 1.4	6.6
PSES (a.u.)	17.8 ± 2.8	18.2 ± 2.2	2.2	18.8 ± 2.5	19.9 ± 2.6 *	5.9	17.9 ± 2.2	18.4 ± 2.9	2.8
PACES (a.u.)	3.8 ± 0.8	4.0 ± 0.6	3.8	3.7 ± 0.6	4.0 ± 0.6 *	8.3	3.5 ± 0.8	3.9 ± 0.8 *	10.5

ML = multi-teaching approach emphasizing linear teaching; MNL = multi-teaching approach emphasizing nonlinear pedagogy; C = control (conventional PE teaching by generalists). Data refers to testing procedures at baseline and after 12 weeks of intervention. ∆% = percentage of within-group post-intervention changes compared to baseline. “a.u.” = arbitrary units. Significant values: * *p* < 0.05.

**Table 2:** Between-group comparisons of teaching approach on critical thinking, executive functioning, physical activity, self-efficacy, and enjoyment.

Variables	Kruskal-Wallis	Dunn’s Post-Hoc			ES
		MLI/MNL	MLI/C	MNL/C	
DMA Locomotor task *(§)*	0.000	0.083	0.003	0.000	0.169 (*medium*)
DMA Stability task	0.012	1.000	0.030	0.030	0.048 (*small*)
DMA Manipulative task	0.000	0.459	0.000	0.000	0.230 (*medium*)
RNG task	0.001	0.147	0.176	0.000	0.122 (*medium*)
RNG task Errors *(§)*	0.000	0.028	0.022	0.000	0.228 (*medium*)
PAQ-C	0.726				
PSES	0.006	0.023	1.000	0.014	0.083 (*medium*)
PACES	0.947				0.001 (*very small*)

ML = multi-teaching approach emphasizing linear teaching; MNL = multi-teaching approach emphasizing nonlinear pedagogy; C = control (conventional PE teaching by generalists). *p* values refer to the Kruskal–Wallis test and Dunn’s post-hoc comparisons between teaching approaches on data collected after 12 weeks of intervention. *(§)* = differs from baseline ML and C (*p* < 0.05). When variables differed at baseline (DMA locomotor task and RNG task errors), the intervention effects were analyzed with the Kruskal–Wallis analysis of variance and Dunn’s post-hoc comparisons to the Δ values (after-before differences). ES = epsilon squared effect size.

**Table 3:** Duration of activities and percentage differences between teaching approaches, as measured by physical education lesson video analysis with IFITS.

Activity	MLI	MNL	C
Lesson duration (min)	58.0 ± 1.1	57.4 ± 2.4	43.4 ± 10.5
Resting time (%)	4.3	8.5	28.3
In action time (%)	83.7	74.3	71.7
Reflection time (%)	12.0	17.2	0.0
*Teaching style*			
Linear (%)	82.2	20.5	23.8 (a)
Nonlinear (%)	17.8	79.5	76.2 (b)

ML = multi-teaching approach emphasizing linear teaching; MNL = multi-teaching approach emphasizing nonlinear pedagogy; C = control (conventional PE teaching by generalists). Teaching styles shows the duration of the teachers’ multi-teaching style combination, which was expected to be 80% linear and 20% nonlinear in ML, and 20% linear and 80% nonlinear in MNL. Further details of the teacher’s styles are provided under C: (a) = prescriptive, no imitation; (b) = free play.

## Data Availability

The data presented in this study are available on request from the corresponding author.

## Appendix A

**Table A1:** Table of pedagogical principles guiding enriched Multi-teaching PE programs.

Lessons Plan’s Key Points	
*I*	Promote social interaction with a positive participation among students.
*II*	Stimulate and involve students in activities which are suitable for them (not too difficult, not too easy).
*III*	Increase motor competence by involving opportunities for motor learning and fitness improvement during each activity and by providing information and engaging in reflection about physical exercise usefulness and their practice outside the school context.
*IV*	Develop a positive, fun, and enjoyable setting based on satisfaction in improving and learning new skills.
*V*	Experience a pleasant experience during PE lessons as a result of a positive children’s engagement and commitment (mastery climate)

## Appendix B

**Table A2:** An example of the structured practice prevalently based on linear pedagogy, as used in teaching ML.

Linear Pedagogy Approach: “Long Live the Gymnastic.”
The traditional approach is based on the following principles: - Age-related exercises (“the age of fantasy,” which consider participants’ motor ability level - Systematic principles (from more comfortable to more complex exercises) - Continuity of the purpose (mainly in a block of content) - Based on reproductive teaching styles - Based on imitative exercising in which fantasy is used to emulate objects or movements proposed by the teacher and reflective action. The approach is centered on developing proficiency and is oriented on education to the movement and proficiency barrier concept.
*Example of postural exercises—Stability*
“The Sunflower” The teacher says: “Who wants to be a sunflower? - The sunflower is a big flower that turns following the sun! - Look at me! I am planted here. I can only move my stem to look at the sun because it allows me to grow up, and I love it! - Look! Look! The sunrise over there! The sun rises higher and higher in the sky.” The children imitate the teacher’s movements, turning the stem to the right, backward, to the right, and then forward. “The sun reaches the higher point and then starts going down with the sunset - Now close your eyes, here comes the night - Look! A new day is coming!” and so on. *Identifying criteria of linear pedagogy:* The above-described examples are imitative command exercises; the teacher acts as a model showing the exercise’s correct form. The purpose is to apply a prescriptive method (everyone does the same exercise).
*Example of a dynamic exercise—Locomotor*
“The soldier’s march.” Walking is proposed. It is the fundamental motor pattern of kinetics, human movement, and “good posture.” Children are first requested to freely walk through the gym, stopping at the stop signal with the forehead turned to the teacher, wherever they are. Creating a story, the teacher beats a rhythm with the tambourine, stimulating the pace to follow while walking. When the tambourine stops, children stop walking and turn their foreheads to the teacher. Children are invited to repeat the rhythm by clapping their hands or with their voices. The teacher gives an example and asks the children to walk, respecting the rhythm. The exercise is repeated until all children understand the exercise. The rhythmic guide can later be varied by accelerating, slowing down, or syncopating it. Children are invited to make the rhythm to guide their mates performing the exercise. During break times, the teacher gives brief instructions about placing the feet correctly, how to swing arms, or maintain the trunk’s correct position. *Identifying criteria of linear pedagogy:* Command and reciprocity reproduction styles are used. The purpose is prescriptive (everyone follows the same rhythm).
*Example of manipulative exercises—object control*
“The teacher ball.” The children are in a semicircle; the teacher is 3–4 m in front of them. The teacher throws the ball to the first player starting from the right side. The child must catch the ball as a goalkeeper (forearms, chest), as previously demonstrated by the teacher. After the catch, the child must give the ball back to the teacher. The game continues until all children have performed the catch, and everyone entirely understands the game. Then, the class is divided into groups, and the children take turns emulating the teacher’s role. *Identifying criteria of linear pedagogy:* A command reproduction style is used. A ball catch model is proposed following systematic and adequacy criteria (nerve maturation and fiber myelination move from the body to limbs). A more comfortable ball catch is proposed (forearms, chest).

## Appendix C

**Table A3:** An example of the structured practice prevalently based on nonlinear pedagogy, as used in teaching MNL.

Nonlinear Pedagogy Approach: “Once upon a Time…the Gymnastics.”
Based on an active pedagogy, children become the protagonist of their education. Pedagogy is based on the following criteria: - The teacher does not plan exercises but supports the student in independent research of motor solutions. - Randomized and varied activities are proposed (environment, body, and task variations) without indicating exercise models. - Experiences that educate through movement based on production styles and hologrammatic principle. - The focus is not set on the executive quality of movement but on creating global “motor backgrounds” through trial learning. Developing the child’s creativity is a main focus.
*Example of postural exercises—Stability*
“Now, let’s try to discover how to play with our body by doing movement staying on-site - Who can invent exercises while staying on-site? - Fabio, try staying in balance with one leg like a crane - Who can invent new methods to stay in balance? - Which other parts of your body can you use to stay in balance? - Look at Luca: he has discovered he can stay in balance with the butt - Who can stay in balance with the support of three parts of their body? - What about trying on the gymnastic mattress or on a line? - Can you stay in pairs while standing only on two feet and two hands? - How can you do that? - Can you invent a different method to stay on-site in pairs? - Is it possible to maintain a standing position? How? In a sitting position? If you stay in all four like some animals, can you move the back? - Giovanni has proposed an exercise; who can tell me how or why is it useful? Which part of his body did he move? Who can repeat the exercise with the eyes closed? - Is it easier? Is it more difficult? Why?” Children are divided into groups; each group has to invent exercises to move arms, back, and legs. Older children can be requested to find exercises ordered from the easiest to the most difficult. In the end, each group presents the exercises it designed. *Identifying criteria of nonlinear pedagogy*: Production styles based on guided discovery and problem-solving are used, but no specific exercises. Several variations can be proposed concerning the use of the body (e.g., involving different limbs, changing sensory feedback), environment (e.g., changing teammates, performing on different surfaces), and task (e.g., changing the nature of the exercises—postural or balance).
*Example of a dynamic exercise—Locomotor*
Children are free to move in any direction in a defined space without colliding. “How many ways can we move in? - Who can invent new methods to move? - How can we do our steps while walking? - Are Luigi’s steps long? Are they short?”. The teacher simulates a wall by his arms to reduce to half the space available to move. “Can we do the same things in half of the hall? - Can we avoid colliding while running? Is it better to walk? - Are long steps better than short steps? - Do horses move like a human? - How do horses move? - Who can invent new ways to move in all four? - Discover new ways of moving while playing with a mate - Which pair can proceed at the same speed? - Who can reproduce the shadow as his teammate - Where is your shadow? Is it in front of or behind you?” *Identifying nonlinear pedagogy criteria:* Production styles are used based on free exploration (everyone does what they want), guided discovery (searching different ways to move around), and problem-solving (understanding which movements are better to do in a reduced or a widened area). Demonstrations are not provided. Variations in how to use the body (e.g., by short, long, slow, and fast steps), environment (e.g., an extended or reduced area; together with mates), task (e.g., by a different type of movement) are suggested.
*Example of manipulative exercises* *—object control*
Children sit down in a circle. The teacher gives one ball to each child and says: “As I give you the ball, you can stand up and use it as you wish.” Children move around the gym by bouncing, hitting, and throwing the ball. The teacher raises one arm to get attention. “Let’s have a look at what Luigi is doing. How did he touch the ball? With the hands! - How many things can we do with our hands? - You all have to invent five different things at least that you can do with your hands”. A pause to reflect is allowed, and when the game continues, several answers will likely be found. “What other parts of your body can you use more than your hands? What can you do with your feet? Which foot is Luigi using? The right or the left one?” Luigi spins on himself. “Can you use the same foot? And the corresponding hand?” Each child exhibits all he learned. The exploration of motor possibilities continues by using different objects, such as a balloon. *Identifying nonlinear pedagogy criteria:* Production styles are used and are based on individual supervised experiences (free exploration in which everyone can do what they want), guided discovery (guiding in different ways to use a tool), and problem-solving (children have to understand which foot or hand a mate is using and try to use the same body part in the game). No demonstrations are provided. Variations are proposed in the body and environment use (e.g., changing the body part or the objects used while acting).
